# Pharmacist Segments Identified from 2009, 2014, and 2019 National Pharmacist Workforce Surveys: Implications for Pharmacy Organizations and Personnel

**DOI:** 10.3390/pharmacy8020049

**Published:** 2020-03-26

**Authors:** Jon Schommer, William Doucette, Matthew Witry, Vibhuti Arya, Brianne Bakken, Caroline Gaither, David Kreling, David Mott

**Affiliations:** 1 College of Pharmacy, University of Minnesota, 308 Harvard Street, S.E., Minneapolis, MN 55455, USA; cgaither@umn.edu; 2 College of Pharmacy, University of Iowa, S518 PHAR, Iowa City, IA 52242, USA; william-doucette@uiowa.edu (W.D.); matthew-witry@uiowa.edu (M.W.); 3 College of Pharmacy and Health Sciences, St. John’s University, St. Augustine Hall, B48, Queens, NY 11439, USA; aryav@stjohns.edu; 4 School of Pharmacy, Medical College of Wisconsin, Health Research Center, 8701 Watertown Plank Rd, Milwaukee, WI 53226, USA; bbakken@mcw.edu; 5 School of Pharmacy, University of Wisconsin – Madison, 777 Highland Avenue, Madison, WI 53705, USA; david.kreling@wisc.edu (D.K.); david.mott@wisc.edu (D.M.)

**Keywords:** pharmacist, workforce, dispensing, patient care, trends, support personnel

## Abstract

**Background/Objective:** Findings from the 2009 and 2014 National Pharmacist Workforce Surveys showed that approximately 40% of U.S. pharmacists devoted their time primarily to medication providing, 40% contributed a significant portion of their time to patient care service provision, and the remaining 20% contributed most of their time to other health-system improvement activities. The objective of this study was to characterize the U.S. pharmacist workforce into segments based on the proportion of time they spend in medication providing and patient care services and compare changes in these segments between 2009, 2014, and 2019. **Methods:** Data from 2009, 2014, and 2019 National Pharmacist Workforce Surveys were analyzed. Responses from 1200 pharmacists in 2009, 1382 in 2014, and 4766 in 2019 were used for analysis. Respondents working in the pharmacy or pharmacy-related fields reported both their percent time devoted to medication providing and to patient care services. Medication providing included preparing, distributing, and administering medication products, including associated professional services. Patient care services were professional services designed for assessing and evaluating medication-related needs, monitoring and adjusting patient’s treatments, and other services designed for patient care. For each year of data, pharmacist segments were identified using a two-step cluster analysis. Descriptive statistics were used for describing the characteristics of the segments. **Results:** For each year, five segments of pharmacists were identified. The proportions of pharmacists in each segment for the three surveys (2009, 2014, 2019) were: (1) medication providers (41%, 40%, 34%), (2) medication providers who also provide patient care (25%, 22%, 25%), (3) other activity pharmacists (16%, 18%, 14%), (4) patient care providers who also provide medication (12%, 13%, 15%), and (5) patient care providers (6%, 7%, 12%). In 2019, other activity pharmacists worked over 45 hours per week, on average, with 12 of these hours worked remotely. Patient care providers worked 41 hours per week, on average, with six of these hours worked remotely. Medication providers worked less than 40 hours per week, on average, with just one of these hours worked remotely. Regarding the number of patients with whom a respondent interacted on a typical day, medication providers reported 18 per day, patient care providers reported 11 per day, and other activity pharmacists reported 6 per day. In 2009, 8% of patient care providers worked in a setting that was not licensed as a pharmacy. In 2019, this grew to 17%. **Implications/Conclusions**: The 2019 findings showed that 34% of U.S. pharmacists devoted their time primarily to medication providing (compared to 40% in 2009 and 2014), 52% contributed a significant portion of their time to patient care service provision (compared to 40% in 2009 and 2014), and the remaining 14% contributed most of their time to other health-system improvement activities. Distinguishing characteristics of the segments suggested that recent growth in the pharmacist workforce has been in the patient care services, with more being provided through remote means in organizations that are not licensed as pharmacies. The findings have implications for pharmacist training, continuing education, labor monitoring, regulations, work systems, and process designs. These changes will create new roles and tasks for pharmacy organizations and personnel that will be needed to support emerging patient care services provided by pharmacists.

## 1. Introduction

Findings from the 2009 and 2014 National Pharmacist Workforce Surveys in the United States revealed five segments of pharmacists: (1) medication providers, (2) medication providers who also provide patient care, (3) other activity pharmacists, (4) patient care providers who also provide medication, and (5) patient care providers [1,2]. The findings from 2009 and 2014 showed similar patterns with approximately 40% of U.S. pharmacists devoted primarily to medication providing, 40% contributing a significant portion of their time (typically, 20% or more) to patient care service provision, and the remaining 20% contributing most of their time to business/organization management, research, education, and other health-system improvement activities.

**Figure 1:**
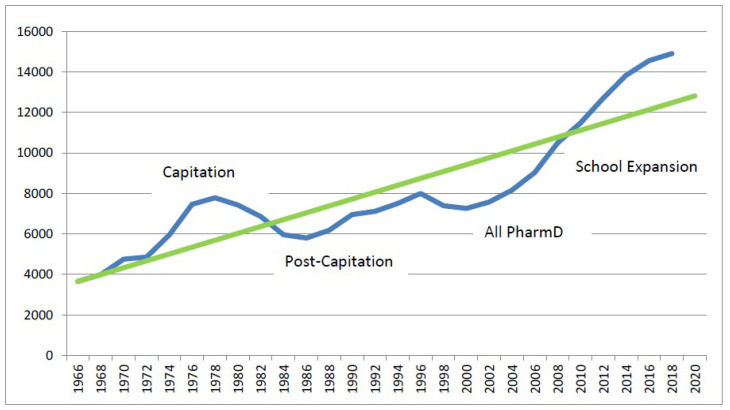
Number of Pharmacist First Professional Degrees by Year of Graduation (1965–2018) with Trend Line. Source: 2017-2018 Profile of Pharmacy Students – American Association of Colleges of Pharmacy, AACP.

The findings from 2009 and 2014 suggested that there remained a need for, and segment of, pharmacists devoted to specialty practices, dispensing, and patient care services, which are delivered at the point-of-care [2]. At that time, increases in the number of pharmacy graduates per year helped the pharmacy profession meet medication provision needs while, at the same time, expand capacity for new roles in patient care [2]. However, the relatively large cohort of pharmacists trained in the 1970s (capitation years) was retiring at this same time [3], and their contributions needed to be replaced (see Figure 1). Consequently, there still was not a substantial surplus of pharmacists that could have been engaged in more intense advancement of pharmacists’ patient care service provision [4]. A recent commentary by Lebovitz and Eddington [5] pointed out that, although pharmacist training focused on clinical knowledge and increased student enrollment during those years, employment in more patient-focused jobs had been minimal. 

Since the time the 2009 and 2014 workforce surveys were conducted, considerable shifts in health services and pharmacist roles in the United States have occurred. For example, the pharmacy profession now performs two distinct types of activities: (1) medicine access and supply, and (2) pharmaceutical care [6]. Work system and process designs for medicine access and supply respond to formal requests from prescribers to supply products and associated services as instructed. In contrast, pharmaceutical care involves work systems and processes that focus on decision-making about medicines therapy and planned consultations between pharmacists, prescribers, and patients that facilitate the aim of improving health outcomes [6]. Baines and colleagues described a “blended pharmacy practice” work system and process design that currently is being used as the pharmacy profession attempts to fulfill both types of activities, often in the same location. 

Furthermore, the pharmacy profession in the United States is close to gaining provider status, which would provide Medicare coverage for certain pharmacist services in health professional shortage areas or medically underserved areas [5]. Also, changes in laws allowing immunizations, medication therapy management, and collaborative practice agreements are opening up patient-focused jobs for pharmacists. Frogner and colleagues [7] proposed that health care delivery overall is being reorganized to achieve greater value, improve access, integrate care among settings, advance population health, and address social determinants of health. To accomplish this, there is a need for telehealth, the application of digital technology, team-based care, and community-based delivery models [7]. In their commentary, Frogner and colleagues specifically mentioned pharmacists as playing integral roles and the need for changes in their scope-of-practice regulations [7]. 

Recent market-driven shifts have moved community pharmacy practice from the traditional “locational convenience” strategy to one in which pharmacies are “being organized by their capacity to operate as health care access points that provide and are reimbursed for patient care and public health services” [8,9,10,11,12]. Also, health-system pharmacy practice has been changing from largely acute care models to more comprehensive integrated care models [13] through horizontal integration with clinics and medical centers so that medication and medical costs can be combined in risk portfolios and meet pay-for-performance goals [14]. 

Vertical integration is affecting pharmacy practice, as well. Insurance companies, wholesalers, manufacturers, integrated delivery networks, pharmacy benefit management companies, pharmacies, clinics, and medical centers are integrating in order to (1) provide coordinated services at a lower cost, (2) improve access to services, (3) leverage data, and (4) bear financial risk for the health outcomes of patient populations [9,14,15,16,17]. 

A special issue in the journal *Pharmacy* focused on pharmacist services and provides further evidence of recent changes in health services and pharmacist roles. In that special issue, Urick and Meggs described the post-pharmaceutical care era and the shift in focus from product to the patient [18]. Ascione proposed the need for pharmacists to be better team members in newly emerging collaborative care and integrated health systems [19]. Goode and colleagues provided a comprehensive categorization of community pharmacy-based patient care services within medication optimization, wellness and prevention screenings, risk assessments, chronic care management, acute care management, patient education, care transitions, and public health domains [20]. Other articles in the special issue further described innovative organizational collaboration [21,22], comprehensive medication management [23,24], transitions of care [25,26], public health initiatives [27,28,29], and tailored patient-centered care and assessment [30,31,32,33]. These are just some examples of the changes in health services and pharmacist roles.

To help make these transitions, significant changes in work systems and processes are being developed, including (1) tech-check-tech processes [34,35,36], (2) patient-tailored packaging and delivery [37], and (3) technological advances [37]. It appears that the “blended pharmacy practice” work system and the process design described by Baines and colleagues [6] continue to evolve. New ways of delivering products, managing inventory, and reimbursing for product costs are being developed. At the same time, new ways for recruiting and connecting patients with practitioners, achieving patient outcomes, organizing space for patients to receive services, and being reimbursed for value-based outcomes are being developed. These significant changes are likely to influence the types of work activities performed by pharmacists and the time they devote to these activities [2]. This, in turn, will necessitate changes for pharmacy workforce support personnel as they augment the roles that pharmacists and pharmacies will serve in health care.

In light of the expansion of pharmacist roles and congruent changes in systems of care provision, our goal was to repeat the segment analyses conducted in 2009 [1] and 2014 [2] using data from the 2019 National Pharmacist Workforce Survey [38]. As was done in 2009 and 2014, the segmentation analysis was based upon pharmacists’ time devoted to medication providing (their traditional role) and to patient care services (their emergent role). A segmentation approach identified key clusters (segments) of the pharmacist workforce and provided a description of their characteristics so that projections could be made regarding future pharmacy profession capacity as cohorts of pharmacists exit the workforce and newly trained pharmacists join the workforce. In addition, the findings were interpreted within the context of the future scope of practice changes that could affect roles filled by pharmacists and pharmacy workforce support personnel. 

## 2. Study Objectives

The overall goal for this study was to repeat the segment analysis of the pharmacist workforce conducted in 2009 [1] and 2014 [2] using data from the 2019 National Pharmacist Workforce Survey. The objectives were to:

1. Identify segments of pharmacists based upon time spent in medication providing and patient care services.
2. Describe segments according to demographic characteristics.
3. Describe segments according to work contributions.
4. Describe segments by work setting.
5. Describe segments according to work activities.
6. Describe year of licensure cohorts to identify trends that might impact future pharmacist capacity for contributing to the U.S. health care system.
7. Compare the findings from the 2019 data with findings from the 2009 and 2014 data.
8. Interpret the findings within the context of future scope of practice changes that could affect roles filled by pharmacists and pharmacy workforce support personnel.

## 3. Methods

Data from 2009, 2014, and 2019 National Pharmacist Workforce Surveys were analyzed [38,39,40]. Data in 2009 and 2014 were collected using a mailed questionnaire to a random sample of licensed pharmacists (3000 in 2009 and 5200 in 2014) obtained from a national data warehouse. In 2019, an electronic survey of 96,100 licensed pharmacists obtained from the National Association of Boards of Pharmacy Foundation was used. Responses from 1200 pharmacists in 2009, 1382 in 2014, and 4766 in 2019 were used for analysis. Two continuous variables were the primary focus of this study: (1) percent time spent in medication providing and (2) percent time spent in the patient care services at each respondent’s primary place of employment. Respondents reported the proportion of time they spent in each of the activities. These were two of the six work activities included in each survey, which were defined as:

**Medication providing**: professional services associated with preparing, distributing, and administering medication products, including associated consultation, interacting with patients about the selection and use of over-the-counter products, and interactions with other professionals during the medication dispensing process.
**Patient care services**: professional services not associated with medication dispensing for assessing and evaluating patient medication-related needs, monitoring and adjusting patients’ treatments to attain desired outcomes, and other services designed for patient care.
**Business/organization management**: managing personnel, finances, and operations.
**Research/scholarship**: discovery, development, and evaluation of products, services, and/or ideas.
**Education**: teaching, precepting, and mentoring of students/trainees/technicians.
**Other**: any activities not described in the above categories.

Data were extracted from each database and analyzed for this report. Two variables (percent time in medication providing and percent time in the patient care services) were utilized for conducting a two-step cluster analysis, with IBM SPSS version 24.0 statistical software (IBM Corp., Armonk, NY, USA). The two-step cluster analysis uses a scalable cluster algorithm. The first step of the analysis is to ‘pre-cluster’ each case (a record) into many small sub-clusters through a sequential clustering approach. The second step of the analysis is to ‘cluster the sub-clusters’ resulting from step one into the final cluster solution using an agglomerative hierarchical clustering method. The log-likelihood distance measure (a probability-based distance) is applied for each step of the analysis so that both continuous and categorical variables can be used if so desired [41].

For inclusion in cluster analysis, respondents needed to report both their percent time devoted to medication providing and to patient care services. Respondents who reported that they were: (1) retired, do not practice pharmacy at all, (2) employed in a career not related to pharmacy, or (3) unemployed were not asked the work activity questions and, thus, not included for analysis. Respondents who were included for analysis were those who reported that they were: (1) practicing as a pharmacist, (2) employed in a pharmacy-related field or position, or (3) retired, but still working in a pharmacy or employed part-time as a pharmacist.

Our primary goal was to identify pharmacist segments and describe them using descriptive statistics within the context of the new roles for pharmacists and new work systems that were mentioned in the introduction of this paper. Thus, after pharmacist segments were identified, they were compared across several demographic variables using Chi-Square and Analysis of Variance statistics. 

## 4. Results

**Figure 2:**
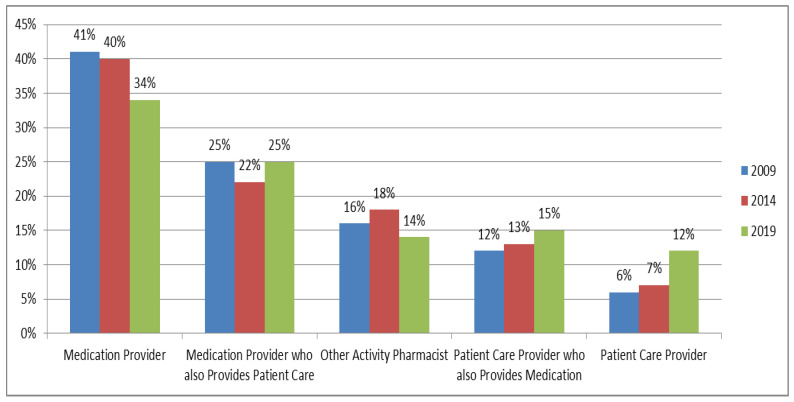
The proportion of U.S. Pharmacists by Segment 2009, 2014, 2019.

Responses from 1200 pharmacists in 2009, 1382 in 2014, and 4766 in 2019 were used for analysis. Cluster analysis identified five segments of pharmacists that we labeled as (1) medication provider, (2) medication provider who also provides patient care, (3) other activity pharmacists, (4) patient care provider who also provides medication, and (5) patient care provider. Figure 2 shows the proportion of pharmacists in each of the five segments, and Table 1 provides a description of each segment in terms of time devoted to medication providing and patient care services. 

Table 2 provides summary comparisons among the five segments in terms of (1) demographic characteristics, (2) work contributions, (3) work settings by column %, (4) work settings by row %, and (5) time currently spent in work activities.

**Medication providing**: professional services associated with preparing, distributing, and administering medication products, including associated consultation, interacting with patients about the selection and use of over-the-counter products, and interactions with other professionals during the medication dispensing process.
**Patient care services**: professional services not associated with medication dispensing for assessing and evaluating patient medication-related needs, monitoring and adjusting patients’ treatments to attain desired outcomes, and other services designed for patient care.

### 4.1. Medication Providers

In our study, 41% of pharmacists in 2009, 40% of pharmacists in 2014, and 34% of pharmacists in 2019 who were employed in pharmacy or in a pharmacy-related field were in the medication provider segment. In 2009/2014/2019, these pharmacists devoted an average of 88%/83%/88% of their time to medication providing and only 5%/6%/4% to patient care services, as defined in this study. Table 2 shows that they were the oldest of the five segments in 2009 and 2014, but not in 2019. Also, in 2009 and 2014, they were less likely to be female and hold a PharmD degree compared to other segments. In 2019, this was no longer the case. In all three study years (2009, 2014, and 2019), this segment contributed the fewest hours worked per week of any segment, and relatively few had residency training (see Table 2). This segment of pharmacists primarily worked in community pharmacy practice settings (78% in 2009, 68% in 2014, and 76% in 2019). In 2019, 57% of respondents who worked in community practice settings were identified as being in the “medication provider” segment of pharmacists, which is similar to 61% of the respondents in 2014 and 60% of respondents in 2009. For 2019, three questions were added to the survey and showed that this segment of pharmacists worked an average of 1.0 hour per week from home or remotely, worked at an average of 2.0 locations for their primary employment, and interacted with an average of 17.6 patients per day as a pharmacy care provider (highest among the five segments).

### 4.2. Medication Providers Who also Provide Patient Care

In our study, 25% of pharmacists in 2009, 22% of pharmacists in 2014, and 25% of pharmacists in 2019 who were employed in pharmacy or in a pharmacy-related field were in the medication provider who also provides patient care segment. In 2009/2014/2019, these pharmacists devoted an average of 65%/60%/63% of their time to medication providing and 19%/22%/16% to patient care services, as defined in this study. Table 2 shows that, in 2009, 48% percent of this segment were female, only 17% had a PharmD degree, and only 4% had residency training. In 2014, 59% were female, 48% had a PharmD degree, and 5% had residency training. By 2019, 62% were female, 59% had a PharmD degree, and 6% had residency training. In 2009, 67% of this segment of pharmacists worked in community pharmacy practice settings, 25% in hospital settings, and 7% in other pharmacy settings. In 2019, 64% worked in community pharmacy settings, 21% worked in hospital settings, and 13% worked in other pharmacy settings. For 2019, three questions were added to the survey and showed that this segment of pharmacists worked an average of 1.5 hours per week from home or remotely, worked at an average of 1.8 locations for their primary employment, and interacted with an average of 16.9 patients per day as a pharmacy care provider (second highest among the five segments).

### 4.3. Other Activity Pharmacists

In 2009, 16% of pharmacists who were employed in pharmacy or in a pharmacy-related field were in the other activity pharmacists segment. In 2014, the proportion was 18%, and, in 2019, the proportion was 14%. In 2009/2014/2019, these pharmacists devoted an average of 5%/6%/3% of their time to medication providing and 3%/5%/4% to patient care services, as defined in this study. Most of their time was devoted to other activities, such as business/organization management, research, education, and other health-system improvement activities. Table 2 shows that, in 2009, 40% were female, 42% had a PharmD degree, and 19% had residency training. In 2014, 54% were female, 58% had a PharmD degree, and 27% had residency training. By 2019, 60% were female, 60% had a PharmD degree, and 26% had residency training. This segment contributed the most hours worked per week of any segment in 2009, 2014, and 2019. In 2009, 45% of this segment of pharmacists worked in ‘other, setting not licensed as a pharmacy’, and 30% worked in a hospital setting. This remained consistent through 2019, with 50% of this segment of pharmacists working in ‘other, setting not licensed as a pharmacy’, and 27% working in a hospital setting. For 2019, this segment of pharmacists worked an average of 11.9 hours per week from home or remotely (highest among the five segments), worked at an average of 2.2 locations for their primary employment, and interacted with an average of 5.5 patients per day as a pharmacy care provider (lowest among the five segments).

### 4.4. Patient Care Providers Who also Provide Medication

This segment (12% of pharmacists in 2009, 13% of pharmacists in 2014, and 15% of pharmacists in 2019 who were employed in pharmacy or in a pharmacy-related field) devoted an average of 33%/29%/30% of their time to medication providing and 43%/49%/43% in 2009, 2014, and 2019, respectively, to patient care services, as defined in this study. Table 2 shows that they were the youngest of the five segments, on average, in both 2009 and 2014, and second youngest in 2019. In 2009, 64% were female, 40% had a PharmD degree, and 25% had residency training. In 2014, 66% were female, 59% had a PharmD degree, and 30% had residency training. In 2019, 65% were female, 75% had a PharmD degree, and 28% had residency training. In 2009, the hours worked per week by this segment were below the overall average. In 2014 and 2019, the hours worked per week were above the overall average. In 2009, 54% of this segment of pharmacists worked in hospital settings, 23% worked in community pharmacy practice settings, and 16% worked in ‘other, licensed pharmacy settings’. In 2014, 70% worked in hospital settings, 13% in community settings, and 14% in ‘other, licensed pharmacy settings’. In 2019, 57% worked in hospital settings, 15% in community settings, and 20% in ‘other, licensed pharmacy settings’. For 2019, this segment of pharmacists worked an average of 2.7 hours per week from home or remotely, worked at an average of 1.6 locations for their primary employment, and interacted with an average of 10.4 patients per day as a pharmacy care provider.

### 4.5. Patient Care Providers

In 2009, 6% of pharmacists who were employed in pharmacy or in a pharmacy-related field were in the patient care provider segment. In 2014, the proportion was 7%, and, in 2019, it grew to 12%. In 2009/2014/2019, these pharmacists devoted an average of 5%/5%/5% of their time to medication providing and 82%/84%/81% to patient care services, as defined in this study. Table 2 shows that they were the second youngest of the five segments, on average, in both 2009 and 2014 and the youngest segment in 2019. In 2009, 59% were female, 53% had a PharmD degree, and 26% had residency training. In 2014, 68% were female, 61% had a PharmD degree, and 34% had residency training. In 2019, 74% were female, 81% had a PharmD degree, and 40% had residency training. In 2009, 64% worked in hospital pharmacy practice settings, 27% worked in ‘other, pharmacy settings’, and 8% worked in ‘other, setting non-pharmacy’. In 2014, 49% worked in hospital settings, 36% worked in ‘other, pharmacy settings’, and 14% worked in ‘other, setting non-pharmacy’. In 2019, 47% worked in hospital settings, 34% worked in ‘other, pharmacy settings’, and 17% worked in ‘other, setting non-pharmacy’. For 2019, this segment of pharmacists worked an average of 6.0 hours per week from home or remotely (second highest among the five segments), worked at an average of 1.8 locations for their primary employment, and interacted with an average of 10.9 patients per day as a pharmacy care provider.

### 4.6. Year of Licensure Cohorts

Table 3 summarizes comparisons for U.S. pharmacists by year of licensure cohorts and provides insight regarding future pharmacy profession capacity as cohorts of pharmacists exit the workforce and newly trained pharmacists join the workforce. For example, Table 3 shows that pharmacists who were licensed before 1980 were typically male, not likely to hold a PharmD degree, and not likely to had residency training. This cohort comprised only 8% of the 2019 survey respondents (393 out of 4686). In comparison, pharmacists who were licensed from 2005 onward were much more likely to be female, over 95% held a PharmD degree, and over 20% had residency training in addition to a PharmD. In 2019, this cohort accounted for 49% of the pharmacist respondents (2300 out of 4686). Pharmacists who were licensed between 1980 and 2004 accounted for the remaining 43% of respondents and showed the transition from BSPharm to PharmD training during this time period. 

## 5. Discussion

The 2019 findings showed that 34% of U.S. pharmacists devoted their time primarily to medication providing (compared to 40% in 2009 and 2014), 52% contributed a significant portion of their time to patient care service provision (compared to 40% in 2009 and 2014), and the remaining 14% contributed most of their time to other health-system improvement activities. This is the first time in the modern pharmacy era that over half of all pharmacists (52%) spend considerable amounts of time in patient care service provision that is separate from patient care that accompanies medication providing. In addition, the segment of pharmacists who devote almost all of their time to patient care services, separate from medication providing, had doubled from 6% of pharmacists in 2009 to 12% of pharmacists in 2019. It should be noted that the data collection method for 2019 used an electronic survey, which was different than a mailed questionnaire approach that was used for 2009 and 2014. However, non-response bias was checked for each of the three survey years, and respondents were found to be representative of the overall pharmacist population of interest in terms of geographic distribution, gender, age, and year of the first licensure. Our confidence in the representativeness of each sample for each of the years was high, but this variation in the method should be considered when interpreting the findings. 

These shifts have significant implications for the work system and process designs that will be needed for new ways of delivering products, managing inventory, and reimbursing for the product cost. At the same time, new ways for recruiting and connecting patients with practitioners, achieving patient outcomes, organizing space for patients to receive services, and being reimbursed for value-based outcomes are needed. We suggested that these significant changes in work systems and processes of care are now the most significant influences on the types of work activities performed by pharmacists and the time they devote to these activities [2]. Distinguishing characteristics of the segments suggested that recent growth in the pharmacist workforce has been in the patient care services, with more being provided through remote means in organizations that are not licensed as pharmacies (see Table 2). This not only has implications for the work system and process designs but also for updates that are needed for scope-of-practice regulations. 

One of our goals was to interpret the findings within the context of the future scope of practice changes that could affect roles filled by pharmacists and pharmacy workforce support personnel. Whereas transitions in clinical training (PharmD, Residency) had contributed to increased capacity for pharmacist contributions to the U.S. Health Care System [1,2,5], the 2019 data showed that transitions in work systems and processes of care (including updates for regulation and roles for pharmacy support personnel) are likely necessary for increasing pharmacist contributions to the U.S. Health Care System in the next decade. As mentioned in the introduction of this paper, pharmacies are being organized by their capacity to operate as healthcare access points that provide patient care and public health services. Comprehensive integrated care models are being created through horizontal integration with clinics, medical centers, community centers, and even places of employment [8,9,10,11,12]. Vertical integration between insurance companies, wholesalers, manufacturers, integrated delivery networks, pharmacy benefit management companies, and health care centers are being formed to coordinate services, improve access, leverage data, and bear financial risk for health outcomes of patient populations [9,14,15,16,17]. As these transitions take place, new ideas for (1) tech-check-tech processes [34,35,36], (2) patient-tailored packaging and delivery [37], and (3) application of new technologies [37] are being applied.

As pharmacist work activities continue to evolve in the future, it is likely that pharmacy support personnel work activities will be impacted as well. A systematic review of pharmacy technician participation in support of medication therapy management service provision [42] has shown that they are most commonly provided assistance with medication reconciliation (70%), documentation (41%), and medication therapy review (30%). Actions least likely to be described include personal medication record development (5%), physical assessment (5%), follow-up (2%), and medication action plan development (0%). Another study [43] has shown that pharmacy technicians in the United States are regularly involved in calling prescribers for clarifications of orders, collecting information from patients, documenting pharmacy care in patient records, and calling patients regarding refills. Other tasks that are not regularly performed but for which technicians report that they are very willing to provide include preparing vaccinations for administration, taking orders from physicians over the phone, transferring a prescription to another pharmacy, and conducting medication reconciliation after a patient is discharged from a hospital [43]. That study has identified four work system and process changes that would help facilitate technicians embrace emerging tasks. They are related to adequate staffing, having time to complete additional tasks, classifying technicians based on specialized skills, and helping cope with stress in the work environment [43]. We highlighted these findings to make the suggestion that, as pharmacist work activities change, pharmacy support personnel work activities will change as well. Koehler and Brown reported that pharmacy technicians and other pharmacy support workforce cadres differ globally in terms of supervision, requirements, education systems, and regulations [44]. Similarly, a pharmacy technician stakeholder consensus conference in the United States [45] has shown variation among technicians in the United States and called for more uniform standards for pharmacy support personnel in terms of legal definition/licensing/regulation, education, entry-level competencies, certification, and advanced practice roles. 

As such changes are made within the pharmacy profession, it must be noted that the U.S. Health Care System is filled with perverse incentives, financial pressures, documentation burdens, the pressure to meet production metrics, and a constant specter of litigation that are creating intensely competing drivers that are emotionally and morally exhausting for pharmacists and pharmacy support personnel as they try to deliver the care that their patients need [46,47]. Thus, there is also a need for a focus on training and system change related to work conditions for personnel, patient safety, payment models, organizational designs, wellbeing, and communications within the overall systems of health care. This will take collective action. 

## 6. Limitations

The results and our interpretation of them should be tempered with the limitations of the study. Slightly different methods were used to obtain the data in each of the three data collection years. These differences might account for some of the findings of the current analyses. The results were based on respondents’ self-reports, raising questions regarding the extent to which respondents gave socially desirable responses. Non-response bias was another limitation. It is possible that responders were more interested in the topic we studied or had stronger opinions about the questions we asked than those who chose not to respond. For our analysis, usable data from respondents working in a pharmacy or a pharmacy-related field were used. While our findings were representative of pharmacists working in a pharmacy or a pharmacy-related field, it should be noted that our analysis did not include licensed pharmacists who were outside of these domains (retired, unemployed, or working outside of a pharmacy-related field). Finally, patient care services might vary widely among responders in terms of specific activities and various roles served. This variable should be viewed as a broadly defined one when interpreting the findings.

## 7. Conclusions

The 2019 findings showed that 34% of U.S. pharmacists devoted their time primarily to medication providing (compared to 40% in 2009 and 2014), 52% contributed a significant portion of their time to patient care service provision (compared to 40% in 2009 and 2014), and the remaining 14% contributed most of their time to other health-system improvement activities. Distinguishing characteristics of the segments suggested that recent growth in the pharmacist workforce has been in the patient care services, with more being provided through remote means in organizations that are not licensed as pharmacies. The findings have implications for pharmacist training, continuing education, labor monitoring, regulations, work systems, and process designs. These changes will create new roles and tasks for pharmacy organizations and personnel that will be needed to support emerging patient care services provided by pharmacists.

## Figures and Tables

**Table 1:** Description of Pharmacist Segments.

Pharmacist Segment	Segment Size (% of total)			Mean Time Devoted to Medication Providing			Mean Time Devoted to Patient Care Services		
	2009	2014	2019	2009	2014	2019	2009	2014	2019
1: Medication Provider	n = 496 (41%)	n = 555 (40%)	n = 1627 (34%)	88%	83%	88%	5%	6%	4%
2: Medication Provider who also Provides Patient Care	n = 303 (25%)	n = 301 (22%)	n = 1194 (25%)	65%	60%	63%	19%	22%	16%
3: Other Activity Pharmacists	n = 193 (16%)	n = 247 (18%)	n = 680 (14%)	5%	6%	3%	3%	5%	4%
4: Patient Care Provider who also Provides Medication	n = 142 (12%)	n = 184 (13%)	n = 689 (15%)	33%	29%	30%	43%	49%	43%
5: Patient Care Provider	n = 66 (6%)	n = 99 (7%)	n = 576 (12%)	5%	5%	5%	82%	84%	81%
Total	N = 1200	N = 1382	N = 4766	58%	52%	51%	17%	20%	22%

**Table 2:** Comparison of U.S. Pharmacist Segments.

	Medication Provider	Medication Provider who also Provides Patient Care	Other Activity Pharmacists	Patient Care Provider who also Provides Medication	Patient Care Provider	Overall
**Demographic Characteristics**						
Mean Age (years)						
2009	52.0	50.2	49.2	45.6	47.4	50.1
2014	49.5	47.3	49.5	44.5	45.8	48.1
2019	45.1	44.1	48.2	41.9	41.3	44.4
Gender (% female)						
2009	41%	48%	40%	64%	59%	47%
2014	52%	59%	54%	66%	68%	57%
2019	67%	62%	60%	65%	74%	65%
Mean Year of First Licensure						
2009	1982	1983	1984	1988	1988	1983
2014	1989	1992	1989	1994	1993	1991
2019	2000	2001	1996	2003	2004	2001
Hold PharmD (%)						
2009	17%	17%	42%	40%	53%	26%
2014	43%	48%	58%	59%	61%	50%
2019	57%	59%	60%	75%	81%	63%
Residency Training (%)						
2009	3%	4%	19%	25%	26%	9%
2014	6%	5%	27%	30%	34%	15%
2019	4%	6%	26%	28%	40%	15%
Both PharmD and Residency (%)						
2009	2%	3%	17%	21%	24%	8%
2014	4%	3%	22%	26%	33%	12%
2019	3%	5%	23%	27%	39%	14%
**Work Contributions**						
Practicing as a Pharmacist (%)						
2009	89%	93%	45%	94%	97%	84%
2014	90%	95%	59%	94%	93%	86%
2019	99%	99%	62%	99%	99%	94%
Mean Hours Worked/Week						
2009	35.6	38.0	44.7	37.2	39.8	38.1
2014	37.2	39.5	46.4	40.3	40.8	40.0
2019	37.9	40.6	45.8	41.9	41.3	40.7
Mean Hours per Week Worked from Home or Remotely						
2009	-	-	-	-	-	-
2014	-	-	-	-	-	-
2019	1.0	1.5	11.9	2.7	6.0	3.5
For Primary Employment, Mean Number of Locations Worked at in a Typical Month						
2009	-	-	-	-	-	-
2014	-	-	-	-	-	-
2019	2.0	1.8	2.2	1.6	1.8	1.9
Mean Number of Patients with whom you Interact as a Pharmacy Care Provider on a Typical Day						
2009	-	-	-	-	-	-
2014	-	-	-	-	-	-
2019	17.6	16.9	5.5	10.4	10.9	11.9
**Current Work Setting (Column %)**						
2009 Community Pharmacy ^a^	78%	67%	10%	23%	1%	-
2014 Community Pharmacy ^a^	68%	58%	15%	13%	1%	-
2019 Community Pharmacy ^a^	76%	64%	9%	15%	3%	-
2009 Hospital Setting	15%	25%	30%	54%	64%	-
2014 Hospital Setting	17%	28%	23%	70%	49%	-
2019 Hospital Setting	10%	21%	27%	57%	47%	-
2009 Other, Pharmacy Setting ^b^	7%	7%	15%	16%	27%	-
2014 Other Pharmacy Setting ^b^	14%	14%	16%	14%	36%	
2019 Other Pharmacy Setting ^b^	11%	13%	14%	20%	34%	
2009 Other, Setting Non-Pharmacy ^c^	<1%	1%	45%	7%	8%	-
2014 Other, Setting Non-Pharmacy ^c^	1%	<1%	46%	3%	14%	
2019 Other Setting Non- Pharmacy ^c^	2%	2%	50%	8%	17%	
**Current Work Setting (Row %)**						
2009 Community Pharmacy ^a^	60%	32%	3%	5%	<1%	-
2014 Community Pharmacy ^a^	61%	29%	6%	4%	<1%	
2019 Community Pharmacy ^a^	57%	35%	3%	5%	<1%	
2009 Hospital Setting	23%	24%	17%	23%	13%	-
2014 Hospital Setting	23%	20%	14%	31%	12%	
2019 Hospital Setting	13%	20%	15%	31%	21%	
2009 Other Pharmacy Setting ^b^	29%	16%	23%	18%	14%	-
2014 Other, Pharmacy Setting ^b^	36%	18%	18%	12%	16%	
2019 Other, Pharmacy Setting ^b^	24%	20%	13%	18%	25%	
2009 Other, Setting Non-Pharmacy ^c^	1%	2%	83%	10%	5%	^-^
2014 Other, Setting Non-Pharmacy ^c^	4%	1%	81%	4%	10%	
2019 Other, Setting Non-Pharmacy ^c^	7%	4%	61%	11%	17%	
**Mean % of Time Currently Spent in Work Activities**						
2009 Medication Providing	88%	65%	5%	33%	5%	58%
2014 Medication Providing	83%	60%	6%	29%	5%	52%
2019 Medication Providing	88%	63%	3%	30%	5%	51%
2009 Patient Care Services	5%	19%	3%	43%	82%	17%
2014 Patient Care Services	6%	22%	5%	49%	84%	20%
2019 Patient Care Services	4%	16%	4%	43%	81%	22%
2009 Business/Org. Management	5%	10%	41%	9%	3%	12%
2014 Business/Org. Management	5%	8%	39%	7%	2%	12%
2019 Business/Org. Management	4%	10%	39%	8%	3%	11%
2009 Research	<1%	1%	18%	4%	3%	4%
2014 Research	<1%	1%	15%	3%	2%	4%
2019 Research	<1%	1%	12%	4%	2%	3%
2009 Education	2%	4%	8%	8%	6%	4%
2014 Education	4%	7%	10%	9%	6%	7%
2019 Education	3%	7%	12%	11%	7%	7%
2009 Other ^d^	1%	1%	25%	5%	2%	5%
2014 Other ^d^	1%	2%	24%	3%	2%	6%
2019 Other ^d^	1%	2%	29%	3%	1%	6%

Note: All statistical comparisons (ANOVA, Chi-Square) significant at p < 0.002. ^a^ “Community Pharmacy Practice” included independent, chain, mass merchandiser, and supermarket pharmacies. ^b^ “Other, Pharmacy Setting” included nursing home, long term care, health maintenance organization, nuclear, clinic-based, mail service, central fill, and home health/infusion pharmacies. ^c^ “Other, Setting Non-Pharmacy” included pharmacy benefit administration, academic, government administration, pharmaceutical industry, consulting companies, professional associations, and other organizations that were not licensed as a pharmacy. ^d^ Other include activities, such as computer analysis, audit control, continuing education, grants, committee work, communications, consultation, data analysis, drug information services, formulary management, systems implementation, inspections, investigations, information technology work, manufacturing, marketing, medication safety, meetings, policy work, problem resolution, quality assurance, regulatory issues, and writing.

**Table 3:** Comparison of U.S. Pharmacist Year of Licensure Cohorts in 2009, 2014, and 2019.

Year of Licensure Cohort (Year of First Licensure)	Female Gender	Age (years)	Hold PharmD Degree	Residency Training	Have Both PharmD and Residency	% (Medication Provider)	% (Medication Provider who also Provides Patient Care)	% (Other Activity Pharmacist)	% (Patient Care Provider who also Provides Medication)	% (Patient Care Provider)
**2009 Survey Data**										
2005 to 2006 (n = 23)	70%	30.9	96%	30%	30%	52%	4%	9%	13%	22%
2000 to 2004 (n = 101)	66%	33.7	75%	22%	21%	33%	23%	18%	20%	7%
1995 to 1999 (n = 136)	67%	38.2	46%	13%	13%	31%	27%	18%	19%	5%
1990 to 1994 (n = 142)	66%	42.0	30%	14%	14%	44%	23%	12%	11%	10%
1985 to 1989 (n = 141)	58%	47.0	17%	6%	6%	38%	26%	17%	15%	4%
1980 to 1984 (n =164)	50%	51.2	20%	7%	6%	35%	29%	21%	9%	6%
1975 to 1979 (n = 188)	39%	55.6	12%	6%	3%	47%	23%	16%	9%	5%
1970 to 1974 (n = 133)	22%	60.7	7%	3%	0%	39%	30%	17%	8%	6%
1965 to 1969 (n = 74)	10%	65.4	5%	7%	3%	47%	24%	18%	10%	1%
1960 to 1964 (n = 41)	10%	70.0	8%	3%	3%	71%	20%	7%	2%	0%
Before 1960 (n = 33)	6%	77.1	9%	0%	0%	73%	21%	6%	0%	0%
OVERALL (N = 1176)	47%	51.6	26%	9%	8%	41%	25%	16%	12%	6%
**2014 Survey Data**										
2010 to 2013 (n = 111)	63%	30.5	96%	28%	28%	37%	23%	8%	21%	11%
2005 to 2009 (n = 174)	71%	33.9	95%	22%	22%	35%	24%	15%	17%	9%
2000 to 2004 (n = 153)	71%	38.3	85%	27%	26%	34%	24%	17%	16%	9%
1995 to 1999 (n = 137)	72%	43.7	52%	16%	14%	39%	23%	21%	12%	5%
1990 to 1994 (n = 157)	66%	47.5	31%	13%	11%	41%	19%	20%	15%	5%
1985 to 1989 (n = 147)	61%	52.1	28%	8%	6%	40%	21%	22%	11%	7%
1980 to 1984 (n = 165)	51%	55.9	18%	8%	4%	38%	23%	19%	12%	9%
1975 to 1979 (n = 162)	36%	60.9	18%	10%	2%	48%	19%	20%	9%	6%
1970 to 1974 (n = 73)	21%	65.3	18%	5%	2%	41%	22%	19%	8%	10%
1965 to 1969 (n = 39)	18%	69.7	9%	16%	3%	49%	18%	21%	10%	3%
1960 to 1964 (n = 15)	13%	74.5	0%	0%	0%	80%	13%	7%	0%	0%
Before 1960 (n = 5)	20%	78.0	0%	0%	0%	40%	0%	40%	0%	20%
OVERALL (N = 1338)	60%	48.1	50%	15%	13%	40%	22%	18%	13%	7%
**2019 Survey Data**										
2015 to 2019 (n = 842)	68%	30.4	97%	22%	22%	34%	28%	6%	17%	16%
2010 to 2014 (n = 1054)	69%	33.6	98%	22%	22%	32%	25%	11%	18%	14%
2005 to 2009 (n = 404)	75%	39.0	95%	23%	23%	31%	21%	16%	17%	16%
2000 to 2004 (n = 296)	73%	43.4	80%	11%	11%	33%	25%	17%	13%	12%
1995 to 1999 (n = 397)	74%	48.6	43%	9%	9%	39%	26%	12%	14%	9%
1990 to 1994 (n = 515)	70%	52.1	28%	8%	6%	35%	24%	19%	12%	10%
1985 to 1989 (n = 413)	62%	56.2	22%	7%	5%	34%	26%	20%	10%	10%
1980 to 1984 (n = 372)	55%	60.5	15%	10%	5%	35%	24%	19%	12%	10%
1975 to 1979 (n = 245)	37%	65.0	18%	5%	4%	40%	19%	25%	8%	8%
1970 to 1974 (n = 102)	28%	69.9	18%	10%	5%	38%	24%	20%	11%	9%
1965 to 1969 (n = 27)	7%	75.1	11%	4%	0%	37%	22%	19%	11%	11%
1960 to 1964 (n = 13)	31%	76.5	8%	0%	0%	62%	23%	8%	8%	0%
Before 1960 (n = 6)	0%	88.8	17%	17%	17%	17%	17%	17%	17%	33%
OVERALL (N = 4686)	65%	44.4	64%	15%	14%	34%	25%	14%	15%	12%

2009 Survey: N does not total 1200 due to missing data; 2014 Survey: N does not total 1382 due to missing data; 2019 Survey: N does not total 4766 due to missing data.

## References

[1] Schommer J.C., Planas L., Johnson K.A., Doucette W.R., Gaither C.A., Kreling D.H., Mott D.A. (2010). Pharmacist Contributions to the U.S. Health Care System. Innov. Pharm..

[2] Schommer J.C., Gaither C.A., Doucette W.R., Kreling D.H., Mott D.A. (2015). Pharmacist Contributions to the U.S. Health Care System Reported in the 2009 and 2014 National Pharmacist Workforce Surveys. Innov. Pharm..

[3] 2017–2018 Profile of Pharmacy Students—AACP, American Association of Colleges of Pharmacy. www.aacp.org.

[4] Maine L.L. (2019). It Really Isn’t That Simple. Am. J. Pharm. Educ..

[5] Lebovitz L., Eddington N.D. (2019). Trends in the Pharmacist Workforce and Pharmacy Education. Am. J. Pharm. Educ..

[6] Baines D., Bates I., Bader L., Hale C., Schneider P. (2018). Conceptualising production, productivity and technology in pharmacy practice: A novel framework for policy, education and research. Hum. Resour. Health.

[7] Frogner B.K., Fraher E.P., Spetz J., Pittman P., Moore J., Beck A.J., Buerhaus P.I. (2020). Modernizing Scope-of-Practice Regulations—Time to Prioritize Patients. N. Engl. J. Med..

[8] Olson A.W., Schommer J.C., Hadsall R.S. (2018). A 15 Year Ecological Comparison for the Market Dynamics of Minnesota Community Pharmacies from 2002 to 2017. Pharmacy.

[9] Schommer J.C., Olson A.W., Isetts B.J. (2019). Transforming community-based pharmacy practice through financially sustainable centers for health and personal care. J. Am. Pharm. Assoc..

[10] Schommer J.C., Doucette W.R., Johnson K.A., Planas L. (2012). Positioning and integrating medication therapy management. J. Am. Pharm. Assoc..

[11] Schommer J.C., Doucette W.R., Planas L. (2015). Establishing pathways for access to pharmacist-provided patient care. J. Am. Pharm. Assoc..

[12] Knapp K.K., Olson A.W., Schommer J.C., Gaither C.A., Mott D.A., Doucette W.R. (2019). Retail Clinics Co-Located with Pharmacies: A Delphi Study of Pharmacist Impacts and Recommendations for Optimization. J. Am. Pharm. Assoc..

[13] Pedersen C.A., Schneider P.J., Ganio M.C., Scheckelhoff D.J. (2019). ASHP National Survey of Pharmacy Practice in Hospital Settings: Monitoring and Patient Education—2018. Am. J. Health Syst. Pharm..

[14] Healthcare Financial Management Association (2014). Acquisition and Affiliation Strategies.

[15] Greaney T.L., Richman B.D. (2018). Consolidation in Provider and Insurer Markets: Enforcement Issues and Priorities.

[16] Greaney T.L., Richman B.D. (2018). Promoting Competition in Healthcare Enforcement and Policy: Framing an Active Competition Agenda.

[17] Madara J.L. (2018). The Acquisition of Aetna, Inc. by CVS Health Corporation.

[18] Urick B.Y., Meggs E.V. (2019). Towards a Greater Professional Standing: Evolution of Pharmacy Practice and Education, 1920–2020. Pharmacy.

[19] Ascione F. (2019). Preparing Pharmacists for Collaborative/Integrated Health Settings. Pharmacy.

[20] Goode J.-V.K.R., Owen J.A., Page A., Gatewood S. (2019). Community-Based Pharmacy Practice Innovation and the Role of the Community-Based Pharmacist Practitioner in the United States. Pharmacy.

[21] Doucette W.R. (2019). Innovative Collaboration between a Medical Clinic and a Community Pharmacy: A Case Report. Pharmacy.

[22] Knapp K., Yoshizuka K., Sasaki-Hill D., Caygill-Walsh R. (2019). Co-located Retail Clinics and Pharmacies: An Opportunity to Provide More Primary Care. Pharmacy.

[23] Neves C.D.M., Nascimento M.M.G.D., Silva D., Álvares M., Ramalho-De-Oliveira D. (2019). Clinical Results of Comprehensive Medication Management Services in Primary Care in Belo Horizonte. Pharmacy.

[24] Twigg G., David T., Taylor J. (2019). An Improved Comprehensive Medication Review Process to Assess Healthcare Outcomes in a Rural Independent Community Pharmacy. Pharmacy.

[25] Took R.L., Liu Y., Kuehl P.G. (2019). A Study to Identify Medication-Related Problems and Associated Cost Avoidance by Community Pharmacists during a Comprehensive Medication Review in Patients One Week Post Hospitalization. Pharmacy.

[26] Schullo-Feulner A., Krohn L., Knutson A. (2019). Reducing Medication Therapy Problems in the Transition from Hospital to Home: A Pre- & Post-Discharge Pharmacist Collaboration. Pharmacy.

[27] Liu Y., Guthrie K.D., May J.R., DiDonato K.L. (2019). Community Pharmacist-Provided Wellness and Monitoring Services in an Employee Wellness Program: A Four-Year Summary. Pharmacy.

[28] Abraham O., Morris A. (2019). Opportunities for Outpatient Pharmacy Services for Patients with Cystic Fibrosis: Perceptions of Healthcare Team Members. Pharmacy.

[29] Safitrih L., Perwitasari D.A., Ndoen N., Lestari K. (2019). Health Workers’ Perceptions and Expectations of the Role of the Pharmacist in Emergency Units: A Qualitative Study in Kupang, Indonesia. Pharmacy.

[30] Maes K.A., Ruppanner J.A., Imfeld-Isenegger T.L., Hersberger K.E., Lampert M.L., Boeni F. (2018). Dispensing of Prescribed Medicines in Swiss Community Pharmacies-Observed Counselling Activities. Pharmacy.

[31] Kaae S., Nørgaard L.S., Sporrong S.K., Almarsdottir A.B., Kofoed M., Daysh R.F., Jowkar N. (2019). Patients’, Pharmacy Staff Members’, and Pharmacy Researchers’ Perceptions of Central Elements in Prescription Encounters at the Pharmacy Counter. Pharmacy.

[32] Schindel T.J., Breault R.R., Hughes C.A. (2019). “It Made a Difference to Me”: A Comparative Case Study of Community Pharmacists’ Care Planning Services in Primary Health Care. Pharmacy.

[33] Redmond S., Paterson N., Shoemaker S.J., Ramalho-De-Oliveira D. (2019). Development, Testing and Results of a Patient Medication Experience Documentation Tool for Use in Comprehensive Medication Management Services. Pharmacy.

[34] Frost T.P., Adams A.J. (2017). Pharmacist and Technician Perceptions of Tech-Check-Tech in Community Pharmacy Practice Settings. J. Pharm. Pract..

[35] Miller R.F., Cesarz J., Rough S. (2018). Evaluation of community pharmacy tech-check-tech as a strategy for practice advancement. J. Am. Pharm. Assoc..

[36] Andreski M., Myers M., Gainer K., Pudlo A. (2018). The Iowa new practice model: Advancing technician roles to increase pharmacists’ time to provide patient care services. J. Am. Pharm. Assoc..

[37] Loria K. (2020). A Look Ahead: What to Expect from the Pharmacy Landscape in 2020. Drug Topics. https://www.drugtopics.com/latest/look-ahead-what-expect-pharmacy-landscape-2020.

[38] Doucette W.R., Matthew J.W., Vibhuti Arya B.K., Bakken C.A., Gaither D.H., Kreling D.A., Schommer J.C. (2019). 2019 National Pharmacist Workforce Survey.

[39] Schommer C.J., Doucette W.R., Gaither C.A., Kreling D.H., Mott D.A. (2009). Final Report of the 2009 National Pharmacist Workforce Survey.

[40] Gaither A.C., Schommer J.C., Doucette W.R., Kreling D.H., Mott D.A. (2014). 2014 National Pharmacist Workforce Survey.

[41] Yim O., Ramdeen K.T. (2015). Hierarchical Cluster Analysis: Comparison of Three Linkage Measures and Application to Psychological Data. Quant. Methods Psychol..

[42] Gernant S.A., Nguyen M.-O., Siddiqui S., Schneller M. (2017). Use of pharmacy technicians in elements of medication therapy management delivery: A systematic review. Res. Soc. Adm. Pharm..

[43] Doucette W.R., Schommer J.C. (2018). Pharmacy Technicians’ Willingness to Perform Emerging Tasks in Community Practice. Pharmacy.

[44] Koehler T., Brown A. (2017). A global picture of pharmacy technician and other pharmacy support workforce cadres. Res. Soc. Adm. Pharm..

[45] Zellmer W.A., McAllister E.B., Silvester J.A., Vlasses P.H. (2017). Toward uniform standards for pharmacy technicians: Summary of the 2017 Pharmacy Technician Stakeholder Consensus Conference. Am. J. Health Pharm..

[46] Schommer J.C., Gaither C., Goode J.-V.K.R., Owen J.A., Scime G.M., Skelton J.B., Cernasev A., Hillman L. (2019). Pharmacist and student pharmacist views of professional and personal well-being and resilience. J. Am. Pharm. Assoc..

[47] Desselle S.P., Holmes E.R. (2017). Results of the 2015 National Certified Pharmacy Technician Workforce Survey. Am. J. Health Pharm..

